# Sex differences in intraorgan fat levels and hepatic lipid metabolism: implications for cardiovascular health and remission of type 2 diabetes after dietary weight loss

**DOI:** 10.1007/s00125-021-05583-4

**Published:** 2021-10-16

**Authors:** Aaron Jesuthasan, Sviatlana Zhyzhneuskaya, Carl Peters, Alison C. Barnes, Kieren G. Hollingsworth, Naveed Sattar, Michael E. J. Lean, Roy Taylor, Ahmad H. Al-Mrabeh

**Affiliations:** 1 Magnetic Resonance Centre, Translational and Clinical Research Institute, Newcastle University, Newcastle upon Tyne, UK; 2 Human Nutrition Research Centre, Population Health Sciences Institute, Newcastle University, Newcastle upon Tyne, UK; 3 Institute of Cardiovascular and Medical Science, University of Glasgow, Glasgow, UK; 4 School of Medicine, Dentistry and Nursing, University of Glasgow, Glasgow, UK; 5 Present Address: Centre for Cardiovascular Science, Queen’s Medical Research Institute, University of Edinburgh, Edinburgh, UK

**Keywords:** Abdominal fat, Cardiovascular disease, Hepatic VLDL1-TG, Intraorgan fat, Lipid metabolism, Remission of diabetes, Sex, Type 2 diabetes, Weight loss

## Abstract

**Aims/hypothesis:**

Type 2 diabetes confers a greater relative increase in CVD risk in women compared with men. We examined sex differences in intraorgan fat and hepatic VLDL1-triacylglycerol (VLDL1-TG) export before and after major dietary weight loss.

**Methods:**

A group with type 2 diabetes (*n* = 64, 30 male/34 female) and a group of healthy individuals (*n* = 25, 13 male/12 female) were studied. Intraorgan and visceral fat were quantified by magnetic resonance and VLDL1-TG export by intralipid infusion techniques.

**Results:**

Triacylglycerol content of the liver and pancreas was elevated in people with diabetes with no sex differences (liver 16.4% [9.3–25.0%] in women vs 11.9% [7.0–23.1%] in men, *p* = 0.57, and pancreas 8.3 ± 0.5% vs 8.5 ± 0.4%, *p* = 0.83, respectively). In the absence of diabetes, fat levels in both organs were lower in women than men (1.0% [0.9–1.7%] vs 4.5% [1.9–8.0%], *p* = 0.005, and 4.7 ± 0.4% vs 7.6 ± 0.5%, *p*< 0.0001, respectively). Women with diabetes had higher hepatic VLDL1-TG production rate and plasma VLDL1-TG than healthy women (559.3 ± 32.9 vs 403.2 ± 45.7 mg kg^−1^ day^−1^, *p* = 0.01, and 0.45 [0.26–0.77] vs 0.25 [0.13–0.33] mmol/l, *p* = 0.02), whereas there were no differences in men (548.8 ± 39.8 vs 506.7 ± 29.2 mg kg^−1^ day^−1^, *p* = 0.34, and 0.72 [0.53–1.15] vs 0.50 [0.32–0.68] mmol/l, *p* = 0.26). Weight loss decreased intraorgan fat and VLDL1-TG production rates regardless of sex, and these changes were accompanied by similar rates of diabetes remission (65.4% vs 71.0%) and CVD risk reduction (59.8% vs 41.5%) in women and men, respectively.

**Conclusions/interpretation:**

In type 2 diabetes, women have liver and pancreas fat levels as high as those of men, associated with raised hepatic VLDL1-TG production rates. Dynamics of triacylglycerol turnover differ between sexes in type 2 diabetes and following weight loss. These changes may contribute to the disproportionately raised cardiovascular risk of women with diabetes.

**Graphical abstract:**

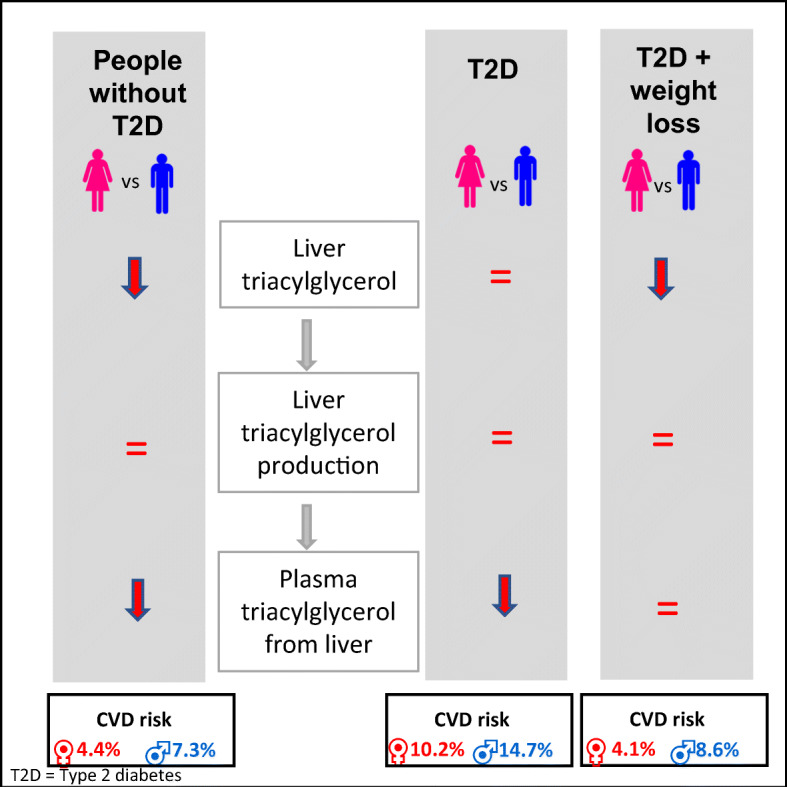

**Supplementary Information:**

The online version contains peer-reviewed but unedited supplementary material available at 10.1007/s00125-021-05583-4.

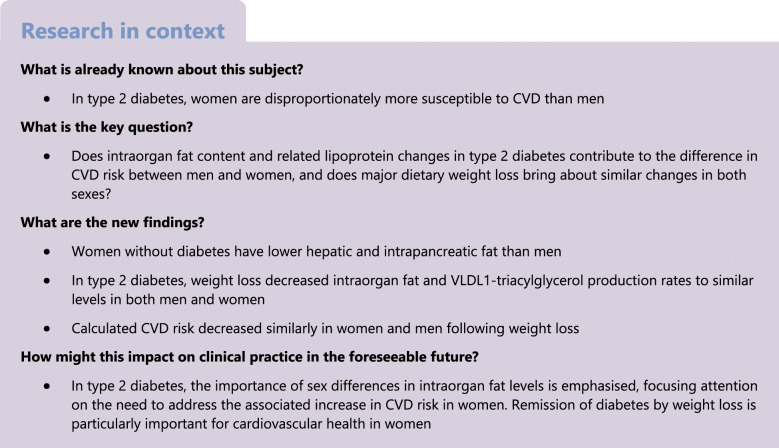



## Introduction

The development of type 2 diabetes causes a disproportionately increased risk of CVD in women, approaching that of men [1]. The higher subcutaneous fat (SAT) in women may normally provide greater safe storage of excess body fat potentially underpinned by a difference in hepatic fat metabolism.

Excess fat accumulation within the liver increases hepatic VLDL1-triacylglycerol (VLDL1-TG) export and plasma VLDL1-TG concentration. We have confirmed this aspect of the twin cycle hypothesis during both remission and redevelopment of diabetes [2]. This hypothesis postulates that type 2 diabetes results from linked vicious cycles leading to excess accumulation of triacylglycerol in the liver and pancreas. Excess VLDL1-TG export will deliver triacylglycerols not only to the pancreas, causing beta cell dysfunction, but also to all sites of ectopic fat accumulation, causing deposition of lipoprotein remnants in coronary and cerebral arteries, increasing CVD risks.

There are few lipid-related studies of women with diabetes, and many studies have overlooked the influence of sex, and the differences underlying the increased CVD of women have not been satisfactorily explained [1, 3]. The primary aim of this study was to quantify the differences in intraorgan fat deposition, hepatic lipoprotein metabolism and CVD risk between women and men with type 2 diabetes and how these variables change after dietary weight loss.

## Methods

### Participants and study design

This study is part of DiRECT (*n* = 64, 30 male/34 female; age, 52.3 ± 1.0 years; BMI, 35.1 ± 0.6 kg/m^2^ [2, 4]). The nondiabetic group (13 male/12 female; age, 55.8 ± 1.2 years; BMI, 29.7 ± 0.8 kg/m^2^) was selected to match the diabetes group after weight loss.

**Fig. 1:**
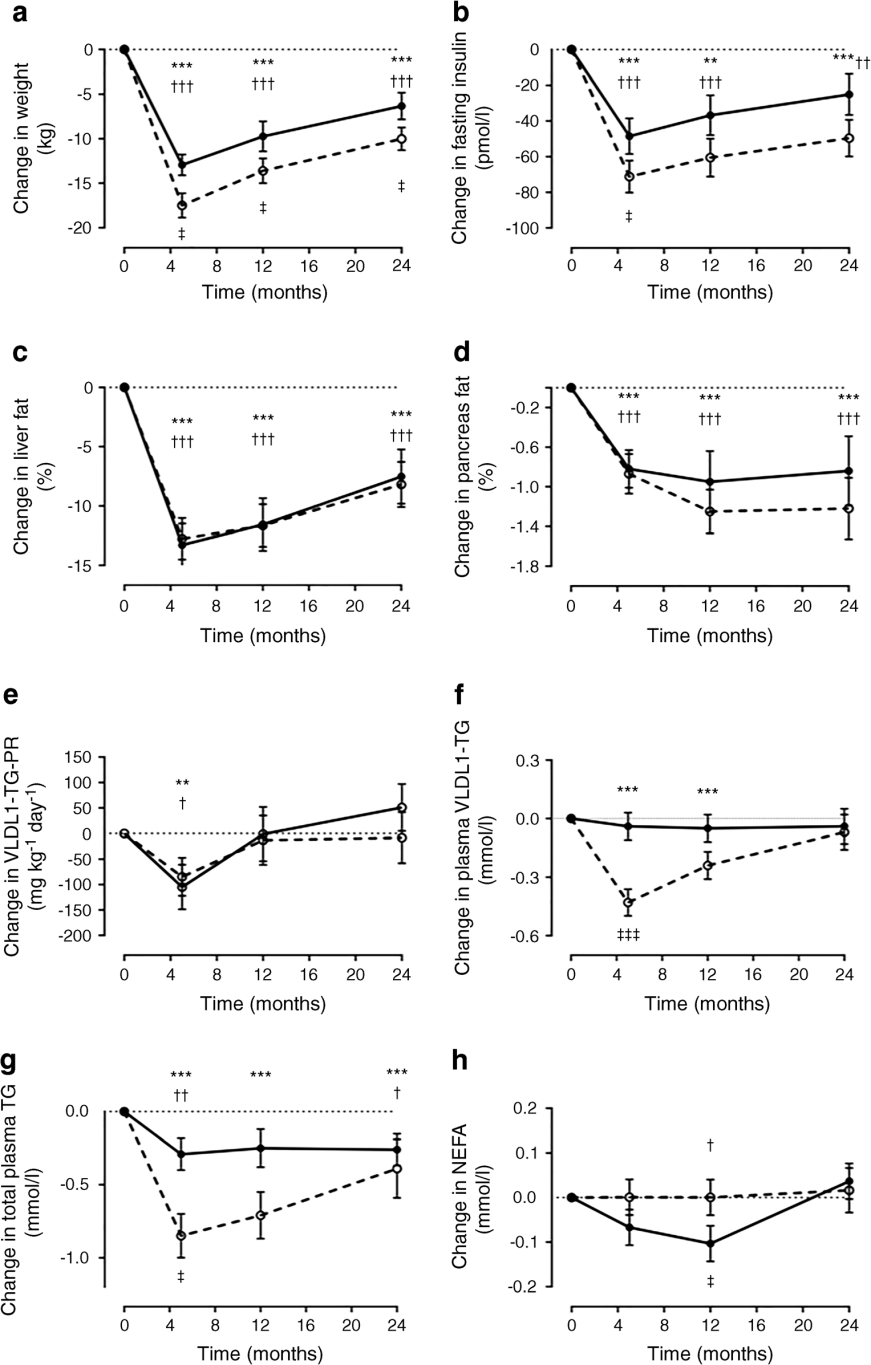
Changes in lipid variables after weight loss in women and men. Changes from the baseline (Δ values) at 5, 12 and 24 months are presented. Statistics within each group reflected the difference between actual values, and statistics between groups were calculated based on the difference in the absolute change at each time point between men and women. Changes in (**a**) body weight, (**b**) fasting plasma insulin, (**c**) liver fat, (**d**) pancreas fat, (**e**) VLDL1-TG production rate, (**f**) plasma VLDL1-TG, (**g**) total plasma TG and (**h**) NEFA are shown for men (dashed line) and women (solid line). Data are presented as mean ± SEM. **p < 0.01, ***p < 0.001 vs baseline (men); †*p* < 0.05, ††*p* < 0.01, †††*p* < 0.001 vs baseline (women); ‡*p* < 0.05, ‡‡‡*p* < 0.001 men vs women. TG, triacylglycerol; VLDL-TG-PR, VLDL-TG production rate

Metabolic studies were carried out at baseline and after dietary weight loss (electronic supplementary material [ESM] Fig. 1).

### Ethics approval

DiRECT is clinically registered (ISRCTN03267836). Ethical approval was obtained from the West of Scotland and Newcastle and North Tyneside Ethics Committees, and written informed consent was obtained from all participants prior to the study.

### Quantification of VLDL1-TG

This was performed using a non-isotopic method [5]. Density gradient ultracentrifugation technique was used to separate the VLDL1 fraction.

### Magnetic resonance studies

Magnetic resonance (MR) data were acquired using a 3.0 Tesla scanner (Philips, the Netherlands). The protocol includes 3-point Dixon acquisition, and MR-opsy/watershed methods for intrapancreatic fat/abdominal fat measurement [6].

### Remission of diabetes

This was defined as HbA_1c_< 6.5% (48.0 mmol/mol) and fasting blood glucose <7.0 mmol/l off any glucose-lowering medication. Relapsers were defined as individuals who lost remission based on the same criteria.

### Biochemical and CVD risk analyses

Triacylglycerol, insulin, glucose and other blood metabolites were measured by standard methods [2]. The QRISK3 (https://qrisk.org/three) was used to assess CVD risk.

### Statistical analysis

The study was powered as described previously [2]. At each time point, the primary outcome is the differences between women and men. Data are presented as mean ± SEM or median and IQR. Student’s *t*, Mann–Whitney *U* or Wilcoxon signed rank tests were employed as appropriate. ANOVA assessed the difference between women and men over time after adjusting for body weight. Minitab and SPSS were used for all statistical analyses. A *p* value < 0.05 was considered statistically significant.

## Results

### Baseline characteristics

Data are presented in Table 1. Women and men were well matched for age, BMI and diabetes duration. Fasting insulin was lower in nondiabetic women than men, and this was not seen in individuals with diabetes. Fasting glucagon was lower in nondiabetic women than men and was similar in diabetes.

**Table 1:** Clinical and metabolic characteristics in men and women at baseline and after weight loss

Characteristic	Baseline (*n* = 64, 34 M/30 F)	5 months (*n* = 57, 31 M/26 F)	12 months (*n* = 48, 27 M/21 F)	24 months (*n* = 45, 25 M/20 F)	ND (*n* = 25, 13 M/12 F)
Age (year)					
Men	53.5 ± 1.2	–	55.1 ± 1.3	56.7 ± 1.3	55.5 ± 1.5
Women	52.3 ± 1.7	–	53.5 ± 2.0	53.9 ± 2.0	56.0 ± 2.0
Diabetes duration (year)					
Men	3.2 ± 0.3	–	4.2 ± 0.3	5.2 ± 0.3	–
Women	2.9 ± 0.3	–	3.9 ± 0.3	4.9 ± 0.3	–
Weight (kg)					
Men	108.1 ± 3.2	90.6 ± 3.0***	93.4 ± 3.3	95.8 ± 3.3	95.6 ± 3.0
Women	92.4 ± 2.3‡‡‡	79.5 ± 2.2***‡‡	81.8 ± 2.9‡	84.7 ± 3.1‡	76.9 ± 3.6‡‡
BMI (kg/m^2^)					
Men	34.7 ± 0.8	29.1 ± 0.8	30.3 ± 0.9	31.1 ± 0.8	30.6 ± 0.8‡‡
Women	35.7 ± 0.9	30.7 ± 0.9	31.7 ± 1.2	31.7 ± 1.2	28.8 ± 1.3‡‡‡
Fasting glucose (mmol/l)					
Men	8.1 (7.3–9.6)†††	6.2 (5.3–6.8)***	5.9 (5.5–7.1)***	6.0 (5.4–6.7)***	5.3 (4.9–5.4)
Women	7.8 (6.6–10.4)†††	6.6 (5.2–7.1)**	6.3 (5.5–7.5)*	6.7 (6.2–9.4)	5.1 (4.8–5.3)
HbA_1c_ (mmol/mol)					
Men	58.0 (54.5–63.0)†††	42.0 (38.0–47.0)***	42.3 (38.2–54.3)***	47.0 (41.0–53.0)**	35.0 (32.0–38.0)
Women	57.0 (48.8–66.0)†††	46.0 (43.0–52.3)**	45.4 (40.3–57.5)**	57.5 (45.0–73.0)	36.0 (34.3–37.3)
HbA_1c_ (%)					
Men	7.5 (7.1–7.9)	6.0 (5.6–6.5)	6.0 (5.6–7.1)	6.5 (5.9–7.0)	5.4 (5.1–5.6)
Women	7.4 (6.6–8.2)	6.4 (6.1–6.9)	6.3 (5.8–7.4)	7.4 (6.3–8.8)	5.4 (5.3–5.6)
Total cholesterol (mmol/l)					
Men	3.9 ± 0.2††	–	3.9 ± 0.2	4.5 ± 0.2*	5.1 ± 0.3
Women	4.7 ± 0.2‡‡	–	4.8 ± 0.3‡‡	5.4 ± 0.3*‡	5.3 ± 0.2
HDL-cholesterol (mmol/l)					
Men	0.96 ± 0.04††	–	1.13 ± 0.06*	1.18 ± 0.05***	1.29 ± 0.09
Women	1.16 ± 0.05‡‡††	–	1.29 ± 0.09	1.40 ± 0.12***	1.57 ± 0.11
Fasting insulin (pmol/l)					
Men	99.5 (54.9–142.0)†††	24.5 (19.1–45.0)***	28.5 (18.6–65.5)***	37.0 (16.0–61.6)***	32.8 (16.2–45.6)
Women	75.3 (55.0–113.0)†††	37.3 (29.4–52.3)***	43.2 (23.0–59.4)***	47.1 (32.7–63.0)**	11.3 (6.1–17.7)‡
Fasting glucagon (pg/ml)					
Men	25.0 (20.5–39.0)†	14.0 (11.0–18.5)***	16.0 (13.0–27.0)**	23.0 (13.0–30.3)*	21.0 (14.0–23.0)
Women	21.0 (17.0–25.5)‡†††	17.0 (11.0–22.5)	16.0 (13.0–25.0)	17.0 (14.0–28.5)	7.5 (5.0–13.3)‡‡
Liver fat (%)					
Men	11.9 (7.0–23.1)†††	1.3 (1.2–1.9)***	1.5 (1.3–2.9)***	4.1 (2.4–6.4)***	4.5 (1.9–8.0)
Women	16.4 (9.3–25.0)†††	1.8 (1.2–6.1)***	3.5 (1.2–9.4)***	8.3 (7.1–13.4)‡‡**	1.0 (0.9–1.7)‡‡
Pancreas fat (%)					
Men	8.5 ± 0.4	7.6 ± 0.4***	7.2 ± 0.4***	7.5 ± 0.4***	7.6 ± 0.5
Women	8.3 ± 0.5†††	7.5 ± 0.5***	7.6 ± 0.4**	7.7 ± 0.6*	4.7 ± 0.4‡‡‡
VLDL1-TG PR (mg kg^−1^ day^−1^)					
Men	548.8 ± 39.8	417.7 ± 28.4**	494.2 ± 42.3*	539.3 ± 37.4	506.7 ± 29.2
Women	559.3 ± 32.9†	474.6 ± 37.0*	548.3 ± 38.7	560.2 ± 38.6	403.2 ± 45.7
Plasma VLDL1-TG (mmol/l)					
Men	0.72 (0.53–1.15)	0.23 (0.15–0.56)***	0.47 (0.25–0.79)*	0.73 (0.37–0.88)	0.50 (0.32–0.68)
Women	0.45 (0.26–0.77)‡†	0.44 (0.23–0.63)	0.35 (0.20–0.75)	0.43 (0.26–0.58)‡	0.25 (0.13–0.33)‡
VLDL1-TG pool (mg)					
Men	2945.5 (1885.4–4772.9)	934.4 (462.5–1735.7)***	1711.7 (711.5–2554.5)**	2309.7 (1180.1–2788.7)	1640.5 (1114.0–2338.8)
Women	1334.1 (920.6–2671.6)‡‡††	1164.6 (466.7–1602.8)	1019.1 (613.9–1616.2)	1181.8 (925.6–1725.5)‡‡	638.9 (293.5–924.00)‡‡
Total TG (mmol/l)					
Men	1.9 (1.3–2.3)	1.1 (0.7–1.5)***	1.1 (0.8–1.6)***	1.2 (0.8–1.7)**	1.5 (1.0–1.60)
Women	1.5 (1.1–2.1)††	1.1 (0.9–1.4)*	1.3 (0.9–1.7)	1.3 (0.9–1.8)	0.9 (0.7–1.2)
Fasting NEFA (mmol/l)					
Men	0.53 ± 0.03	0.53 ± 0.03	0.53 ± 0.03	0.55 ± 0.04	0.57 ± 0.07
Women	0.67 ± 0.04‡‡	0.60 ± 0.04	0.59 ± 0.04*	0.72 ± 0.03‡‡	0.58 ± 0.03
Ketones (mmol/l)					
Men	0.10 (0.10–0.20)†	0.20 (0.10–0.30)*	0.20 (0.15–0.30)*	0.20 (0.18–0.30)*	0.20 (0.20–0.30)
Women	0.20 (0.10–0.28)	0.20 (0.13–0.20)	0.20 (0.20–0.23)	0.20 (0.18–0.23)	0.30 (0.20–0.30)
SAT (cm^2^)					
Men	239.3 ± 15.0	162.0 ± 14.0***	209.6 ± 20.3***	231.8 ± 20.2*	250.3 ± 25.2
Women	397.4 ± 21.8‡‡‡††	318.4 ± 21.9***	341.6 ± 27.4**	350.5 ± 25.2**	279.5 ± 29.2
VAT (cm^2^)					
Men	320.9 ± 12.4	158.1 ± 12.7***	196.6 ± 14.0***	244.8 ± 15.6***	287.3 ± 19.7
Women	226.6 ± 12.3‡‡‡††††	158.2 ± 12.2***	156.6 ± 17.3***	173.1 ± 14.9**	92.8 ± 16.0‡‡‡
QRISK3 (%)					
Men	14.7 (10.0–18.5)††	8.6 (5.9–11.9)***	8.2 (5.8–10.0)***	8.6 (6.8–11.7)*	7.3 (6.5–10.5)
Women	10.2 (7.0–14.8)‡†††	4.1 (2.9–6.7)‡‡***	6.2 (3.8–8.7)***	7.3 (5.3–11.5)*	4.4 (2.9–6.7)‡
Heart age (year)					
Men	67.8 ± 1.2††	60.4 ± 1.3***	59.9 ± 1.3***	62.3 ± 1.6**	59.5 ± 2.1
Women	69.1 ± 1.2†††	59.2 ± 1.7***	62.0 ± 1.8**	63.6 ± 1.8*	57.9 ± 2.0

Paired data with 5 months were presented at baseline (baseline data for those who competed the study was similar to the whole group). Data were presented mean ± SEM or median (IQR) based on distribution

*p < 0.05 vs baseline, **p < 0.01 vs baseline, ***p < 0.001 vs baseline; †*p* < 0.05 vs ND, ††*p* < 0.01 vs ND, †††*p* < 0.001 vs ND; ‡ *p* < 0.05 men vs women, ‡‡ *p* < 0.01 men vs women, ‡‡‡*p* < 0.001 men vs women

F, female; M, male; ND, nondiabetic; TG, triacylglycerol; VLDL1-TG PR, VLDL1-TG production rate

In the nondiabetic group, liver fat in women was 22% that of men (4.5% [1.9–8.0%] vs 1.0% [0.9–1.7%], *p* = 0.005), and this difference was lost in individuals with diabetes (16.4% [9.3–25.0%] vs 11.9% [7.0–23.1%], *p* = 0.57). Similarly, pancreas fat in nondiabetic women was 62% that of men (4.7 ± 0.4% vs 7.6 ± 0.5%, *p*< 0.001) but was almost identical in individuals with diabetes (8.3 ± 0.5% vs 8.5 ± 0.4%, *p* = 0.83).

Compared with the nondiabetic group, women with diabetes had a 39% higher mean VLDL1-TG production rate (559.3 ± 32.9 vs 403.2 ± 45.7 mg kg^−1^ day^−1^, *p* = 0.01), whereas men with those with diabetes had an 8% higher VLDL1-TG production rate (548.8 ± 39.8 vs 506.7 ± 29.2 mg kg^−1^ day^−1^, *p* = 0.40). In the nondiabetic group, VLDL1-TG production rates were numerically but not significantly lower in women than men but almost identical between sexes in individuals with diabetes. Fasting plasma VLDL1-TG levels in nondiabetic women were 50% lower than men (0.25 [0.13–0.33] vs 0.50 [0.32–0.68] mmol/l, respectively, *p* = 0.008), whereas in indivduals with \diabetes, levels in women were 63% of those of men (0.45 [0.26–0.77] vs 0.72 [0.53–1.15] mmol/l, *p* = 0.014. This was reflected in the VLDL1-TG pool size. The fasting plasma NEFA level was identical between women and men in the nondiabetic group but higher in women with diabetes.

There was a major difference in CVD risk between diabetes and nondiabetic groups (Table 1). In the nondiabetic group, women had visceral adipose tissue (VAT) 32% of male values, but it was 71% of male values in women with diabetes. The CVD risk was almost twofold higher in men than women in the nondiabetic group (7.3% [6.5–10.5%] vs 4.4% [2.9–6.7%], *p* = 0.021) and less so in diabetes (14.7% [10.0–18.5%] vs 10.2% [7.0–14.8%], *p* = 0.049). Despite similar actual age, the estimated heart age was higher for both women and men with diabetes (69.1 ± 1.2 vs 57.9 ± 2.0 years, *p*< 0.0001, and 67.8 ± 1.2 vs 59.5 ± 2.1 years, *p* = 0.003, respectively).

### Changes in lipid variables after weight loss

Change from baseline in BMI immediately after weight loss was similar in women and men (−5.0 ± 0.5 vs −5.6 ± 0.4 kg/m^2^, *p* = 0.31) although absolute weight loss was lower in women than men (−12.9 ± 1.2 vs −17.5 ± 1.4 kg, *p* = 0.02, Table 1). Remission of diabetes immediately after weight loss was observed in 65.4% and 71.0% in women and men.

Liver fat decreased similarly in women and men (to 1.8% [1.2–6.1], *p* < 0.0001, and 1.3% [1.2–1.9], *p* < 0.0001, respectively) becoming almost identical to the nondiabetic group.

VLDL1-TG production rate decreased similarly in women and men (to 474.6 ± 37.0, *p* = 0.033, and 417.7 ± 28.4 mg kg^−1^ day^−1^, respectively, *p* = 0.001). However, plasma VLDL1-TG did not change significantly in women (0.45 [0.26–0.77] to 0.44 [0.23–0.63] mmol/l, *p* = 0.92), although it did fall in men (0.72 [0.53–1.15] to 0.23 [0.15–0.56] mmol/l, *p* = 0.0001).

Total triacylglycerol decreased less in women compared with men, and there were no significant changes in fasting NEFA in either group. Pancreas fat and fasting insulin decreased equally in both sexes, but fasting glucagon and ketones levels changed significantly only in men (Table 1).

The decrease in VAT was smaller in women, although the change in SAT was similar to men. CVD risk decreased markedly after weight loss in both sexes (59.8% vs 41.5%) although remained higher in men (Table 1). The estimated heart age decreased, becoming similar to the nondiabetic group (Table 1). CVD risk decreased to a greater extent in those who achieved remission but returned to baseline level in relapsers who gained weight and lost remission (data not shown).

Changes in lipid variables during the 2-year follow-up are shown in Fig. 1.

## Discussion

These data demonstrate clear sex differences in the dynamics of hepatic fat handling in type 2 diabetes. Women normally have lower liver and intrapancreatic fat than men, but these sex differences are lost in diabetes. Women with diabetes have a significantly increased VLDL1-TG production rate and fasting plasma VLDL1-TG concentration compared with nondiabetic women, with smaller differences in men. However, women with diabetes have lower plasma VLDL1-TG than men despite having the same level of liver fat and hepatic VLDL1-TG production rate. Weight loss brought about similar changes in intraorgan fat and VLDL1-TG production rate but not fasting plasma VLDL1-TG levels in both sexes. Estimated CVD risk was high in diabetes, with substantial improvement in both sexes after weight loss [7].

Plasma triacylglycerols were lower in women than men, and the underlying mechanism to explain this difference is not known. In lean nondiabetic individuals, the VLDL1-TG production rate is higher in women than in men, but the reverse is true in people with obesity [8]. In contrast, VLDL1-TG clearance was higher in lean women than men despite their lower rate of VLDL1-TG secretion [9]. We did not find any significant difference in VLDL1-TG production rate between men and women within each group. However, our nondiabetic group represents people who are neither lean nor morbidly obese. Collectively, our data suggest that the VLDL1-TG production rate increases to a greater extent in women who develop diabetes, consistent with a greater filling of intraorgan fat, possibly due to reaching individual limits for SAT storage capacity or change in the biology of adipocyte function.

Most overweight/obese people do not develop type 2 diabetes. This ‘metabolically healthy’ phenotype is genetically determined via alleles associated with higher SAT and lower ectopic fat deposition [10]. Large SAT storage with high expression of lipoprotein lipase (LPL) may increase the uptake of the plasma VLDL1-TG. Stable-isotope studies have shown the larger the SAT in women, the higher the uptake of meal-derived fatty acids with the opposite for VAT [11]. The contribution of de novo lipogenesis to VLDL1-TG synthesis in men increases postprandially, suggesting preferential storage of diet-derived fatty acids in the liver [12]. In women, there is greater diversion of fatty acids towards ketones rather than VLDL1-TG [13]. Taken together, women appear to have more efficient mechanisms to clear excess fat, probably via active uptake and deposition of excess fat within SAT. In our study, women with diabetes had higher NEFA at baseline than men, and differences in regional and plasma LPL activity may underlie more rapid catabolism of plasma VLDL1-TG in women.

Women normally have lower CVD risk, but this increases to a greater extent in type 2 diabetes [1]. In this study, weight loss improved the QRISK3 score substantially, becoming similar to the nondiabetic level in both sexes. Oxidised LDL remnants promote atherosclerosis, and women may be more susceptible to cellular damage at lower levels of circulating lipids. Non-lipid factors could also contribute to women’s increased risk of CVD in diabetes [3]. The underlying mechanisms explaining this sex difference are unclear and unlikely to be affected by menopausal status (ESM Table 1). The observed differences in hepatic fat handling appear relevant. Weight loss was associated with a significant decrease of VLDL1-TG production rate in both sexes but a lesser change in plasma VLDL1-TG and VLDL1-TG pool size in women, possibly a consequence of more rapid conversion of VLDL1 to VLDL2 in women [14].

There are potential limitations of this study: (1) the participants remained substantially overweight despite major weight loss and were not expected to achieve normal metabolism; and (2) the women had a lower weight at baseline, despite similar BMI. However, the sex difference in the VLDL1-TG pool remained significant after adjusting for lean body mass estimated from Boer’s formula [15] or for body weight using ANOVA; (3) we did not measure LPL activity or lipoprotein clearance.

Overall, the data presented here demonstrate major metabolic differences in lipid metabolism between sexes, which underlie differences in cardiovascular outcomes in type 2 diabetes.

## Supplementary Information

ESM (PDF 157 kb)

## Data Availability

Data will be provided on request from the corresponding authors.

## Abbreviations

LPL: Lipoprotein lipase
MR: Magnetic resonance
SAT: Subcutaneous adipose tissue
VAT: Visceral adipose tissue
VLDL1-TG: VLDL1-triacylglycerol
