# Supplementary material for: Sex differences in intraorgan fat levels and hepatic lipid metabolism: implications for cardiovascular health and remission of type 2 diabetes after dietary weight loss
Source: Diabetologia. 2021 Oct 16;65(1):226–33. doi: 10.1007/s00125-021-05583-4 (PMC8660759; doi:10.1007/s00125-021-05583-4)
Supplement: Supplementary file 1 — (PDF 157 kb) [file 125_2021_5583_MOESM1_ESM.pdf]

Electronic Supplementary Materials

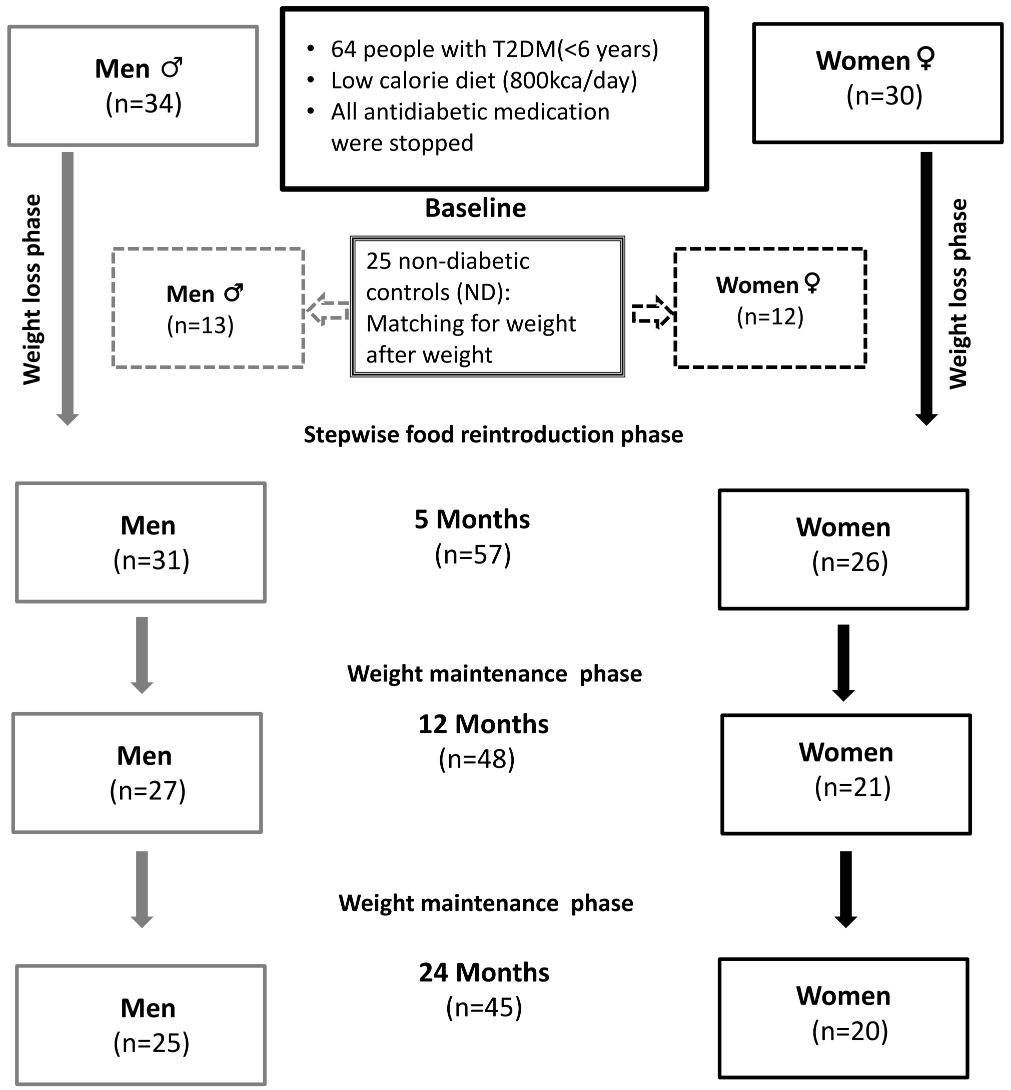

ESM Figure 1. Illustrative diagram showing study design and gender representation within the groups

64 people (34M/30F) with type 2 diabetes (<6 years) randomized within DiRECT were studied at baseline and following 5, 12, and 24 months of commencing the Counterweight-Plus weight management programme: 25 non-diabetic controls (ND: 13M/12F) matched for age and body weight with diabetic group after weight loss, were studied at one single occasion. At 5 months, 6 people withdrew from the study for personal reasons and 1 person did not provide blood sample. At 12 months, 8 other people had left the study for personal reasons, 1 person did not give blood sample). By 24 months, an additional participant had left the study for personal reasons.

ESM Table 1: Comparison of baseline characteristics between women at pre/post-menopausal stage

**Women**	**Baseline** (n=26)
**Age (year)**	
Pre-menopausal (n=11)	44.5±1.4
Post-menopausal (n=15)	58.1±1.6***
**BMI (kg/m^2)**	
Pre-menopausal	36.2±0.6
Post-menopausal	35.4±1.4
**HbA1c (mmol/mol)**	
Pre-menopausal	61.0[49.5-69.5]
Post-menopausal	56.0[49.5-62.0]
**Total Chol. (mmol/l)**	
Pre-menopausal	4.6±0.4
Post-menopausal	4.7±0.3
**HDL Chol. (mmol/l)**	
Pre-menopausal	1.0±0.05
Post-menopausal	1.3±0.08**
**F. Insulin (pmol/l)**	
Pre-menopausal	116.0[90.0-145.5]
Post-menopausal	56.5[50.9-72.2] ***
**Liver Fat (%)**	
Pre-menopausal	14.1[10.9-23.6]
Post-menopausal	16.2[16.4-27.4]
**Pancreas Fat (%)**	
Pre-menopausal	8.5±1.0
Post-menopausal	8.2±0.5
**VLDL1-TG PR. (mg/kg/day)**	
Pre-menopausal	628.5±57.4
Post-menopausal	508.5±34.4
**Plasma VLDL1-TG (mmol/l)**	
Pre-menopausal	0.55[0.30-1.04]
Post-menopausal	0.43[0.25-0.51]
**VLDL1-TG pool (mg)**	
Pre-menopausal	2175.8 [994.4-3127.9]
Post-menopausal	1321.5 [926.3-1536.4]
**Total TG (mmol/l)**	
Pre-menopausal	1.6[1.1-2.3]
Post-menopausal	1.4[1.3-1.9]
**Fasting NEFA (mmol/l)**	
Pre-menopausal	0.61±0.06
Post-menopausal	0.71±0.05
**SAT (cm^2)**	
Pre-menopausal	376.7± 22.2
Post-menopausal	412.6± 34.3
**VAT (cm^2)**	
Pre-menopausal	235.8± 18.2
Post-menopausal	219.9± 17.0

*p<0.05 pre vs. Post-menopausal
**p<0.01 pre vs. Post-menopausal
***p<0.001 pre vs. Post-menopausal
Data are presented as Mean±SEM or Median (IQR)
